# Supplementary material for: Treatment outcomes of multi drug resistant and rifampicin resistant Tuberculosis in Zimbabwe: A cohort analysis of patients initiated on treatment during 2010 to 2015
Source: PLoS One. 2020 Apr 30;15(4):e0230848. doi: 10.1371/journal.pone.0230848 (PMC7192497; doi:10.1371/journal.pone.0230848)
Supplement: Attachment — Submitted filename: RM Authors Response Letter.docx [file pone.0230848.s002.docx]

28 August 2019

PLOS ONE

Dear Editor in Chief

Please find below a reply letter to our submission, titled:

*PONE-D-19-16150: Treatment outcomes of Multi drug resistant and Rifampicin resistant Tuberculosis in Zimbabwe: A cohort analysis of patients initiated on treatment during 2010 to 2015*

We thank the reviewers and the Editor for their valuable comments to our initial submission. We have attempted to address all the queries made. We provide below a point-by-point response to the queries raised and we have also provided both the clean version of the manuscript and a document with track changes.

**Editor comments**

**Comment #1**

1. When submitting your revision, we need you to address these additional requirements.

Please ensure that your manuscript meets PLOS ONE's style requirements, including those for file naming. The PLOS ONE style templates can be found at
<http://www.journals.plos.org/plosone/s/file?id=wjVg/PLOSOne_formatting_sample_main_body.pdf> and <http://www.journals.plos.org/plosone/s/file?id=ba62/PLOSOne_formatting_sample_title_authors_affiliations.pdf>

***Response***

Noted thank you. This has been addressed as per requirement

**Comment #2**

In ethics statement in the manuscript and in the online submission form, please provide additional information about the patient records/samples used in your retrospective study. Specifically, please ensure that you have discussed whether all data/samples were fully anonymized before you accessed them and/or whether the IRB or ethics committee waived the requirement for informed consent. If patients provided informed written consent to have data/samples from their medical records used in research, please include this information.

***Response***

Thank you for raising this very important ethical issue. Data was fully anonymised as only unique identifiers in the form of TB registration numbers were abstracted onto the data collection proforma in place of patient names. The Ethics committee waived the requirement for individual informed consent since this was a retrospective study and the Ministry of Health and Child Care had given permission to access patients clinical records at participating centres.

**Comment #3**

Please note that all PLOS journals ask authors to adhere to our policies for sharing of data and materials: <https://journals.plos.org/plosone/s/data-availability>. According to PLOS ONE’s Data Availability policy, we require that the minimal dataset underlying results reported in the submission must be made immediately and freely available at the time of publication. As such, please remove any instances of 'unpublished data' or 'data not shown' in your manuscript and replace these with either the relevant data (in the form of additional figures, tables or descriptive text, as appropriate), a citation to where the data can be found, or remove altogether any statements supported by data not presented in the manuscript

***Response***

Thank you for raising this. The data not shown on distribution and type of severe adverse events has now been added as Table 3.

**Comment #4**

Our internal editors have looked over your manuscript and determined that it is within the scope of our Antimicrobial Resistance call for papers. This collection of papers is headed by a team of Guest Editors for PLOS ONE: Kathryn Holt (Monash University and London School of Hygiene and Tropical Medicine), Alison H. Holmes (Imperial College London), Alessandro Cassini (WHO Infection Prevention and Control Global Unit), Jaap A. Wagenaar (Utrecht University). The Collection will encompass a diverse range of research articles; additional information can be found on our announcement page: <https://collections.plos.org/s/antimicrobial-resistance>. If you would like your manuscript to be considered for this collection, please let us know in your cover letter and we will ensure that your paper is treated as if you were responding to this call. If you would prefer to remove your manuscript from collection consideration, please specify this in the cover letter.

***Response***

Thank you for considering our manuscript for the PLOS ONE collection on Antimicrobial Resistance. We would like to express our interest to have our manuscript considered as such and we give you explicit permission to process accordingly. We have indicated our interest in the attached cover letter

**Comment #6**

We note that you have indicated that data from this study are available upon request. PLOS only allows data to be available upon request if there are legal or ethical restrictions on sharing data publicly. For information on unacceptable data access restrictions, please see <http://journals.plos.org/plosone/s/data-availability#loc-unacceptable-data-access-restrictions>.
In your revised cover letter, please address the following prompts:
a) If there are ethical or legal restrictions on sharing a de-identified data set, please explain them in detail (e.g., data contain potentially identifying or sensitive patient information) and who has imposed them (e.g., an ethics committee). Please also provide contact information for a data access committee, ethics committee, or other institutional body to which data requests may be sent.

***Response***

Thank you for the comment. Unfortunately we are not permitted to share our dataset due to our local ethics clearance committee and the Ministry of Health and Child Care who are the custodians of all data related to the National TB Programme. We have provided details of institutional heads for both organizations in the cover letter as requested.

**Comment #7**

We note that you have included the phrase “data not shown” in your manuscript. Unfortunately, this does not meet our data sharing requirements. PLOS does not permit references to inaccessible data. We require that authors provide all relevant data within the paper, Supporting Information files, or in an acceptable, public repository. Please add a citation to support this phrase or upload the data that corresponds with these findings to a stable repository (such as Figshare or Dryad) and provide and URLs, DOIs, or accession numbers that may be used to access these data. Or, if the data are not a core part of the research being presented in your study, we ask that you remove the phrase that refers to these data.

***Response***

The data that were not shown have now been added as “Table 3”.

**Additional Editor Comments:**

**Comment #1**

In addition to the Reviewer comments, I have the following comments that the authors should consider.
1. The authors should consider revising their concluding sentence which reads "There is a need for increased uptake of ART". HIV status and ART status was reported at initiation of treatment. Some of the patients who are HIV positive may be newly diagnosed and may not have initiated ART yet - it would be useful to look and see if those who were HIV positive but not on ART at treatment initiation, initiated ART between 2 weeks to 2 months depending on the CD4 count (as per guidelines). You may find some of the HIV positive patients initiated ART after they initiated DR-TB, and therefore it is difficult to make inferences about treatment outcomes.

***Response***

Thank you. In our study, dates on when ART was initiated in relation to commencement of TB treatment were not collected. However, the ART status of those who newly tested HIV-positive are supposed to be updated in the DR-TB register throughout their course of MDR-TB treatment period together with date of ART initiation although the information of ART start dates were missing in our case. As a result this limits our capabilities to determine impact of ART timing on DR-TB treatment outcomes, however we feel there is merit to conclude that ART does improve DR-TB treatment outcomes. Instead we have added to the abstract the following sentence: “Assessing timing of ART initiation in relation to TB treatment outcomes will also require future exploration.” We hope this response is satisfactory.

**Comment #2**

2. Line 120 - I find the use of "short-course" confusing here, since the WHO has guidelines for short- and long-course DR-TB treatment. I would consider rephrasing. The reference [2] "DOTS-Plus and Green Light Committee" refers to 6-8 months with first-line anti-TB treatment.

***Response***

Noted, the term “Short course” has been removed and the sentence now reads as follows: “In 2000, WHO recommended the 20-24 month standardized second-line drug (SLDs) regimens for treatment of MDR/RR-TB patients in resource-limited settings”. We have also removed the reference [2] on the DOTS-Plus and the Green Light Committee.

**Comment #3**

3. Line 134 - requires a reference for "WHO has recommended shorter regimens".

***Response***

Thank you. We have now referenced the 2019 WHO consolidated guidelines on drug-resistant tuberculosis treatment.

**Comment #4**

4. Line 150 - Please clarify what the short course regimen is, in your setting (e.g. shorter, injectable-based, 9-12 month regimen)

***Response***

Noted, thank you. Now clarified and it reads: “In 2016, the short treatment regimen which is over 9-11 months and injectable based was adopted”

**Comment #4 (Duplication of numbering)**

Line 132 - The authors refer to the shorter regimen and Line 152 - authors state that the short regimen has been rolled out in the NTP. Since the paper focuses on the standard long-course RR/MDR-TB regimen (18-24 month), between 2010 - 2015, the authors need to mention why the paper is relevant (since short course has been adopted by the NTP).

***Response***

The short treatment course was piloted since June 2018 and is made available only in a few districts. Even in districts where short regimen is available, very few patients are started on it due to issues in assessing eligibility. Up to now most districts are still implementing the 18-24 month regimen. The short regimen might have been initiated in less than 5% patients. The study is relevant as it informs on the treatment outcomes with longer regimens and also provides a baseline estimate for tracking the successful outcomes rates with scale up of short regimen in future.

**Comment #5**

5. Line 189 - write out "MTB/Rif" the first time it appears in the text; Line 192 - write out "PTB" the first time it appears in the text e.g. pulmonary TB (PTB); Line 224 - write out "PMDT" the first time it appears in the text; Line 230-233 - write out plus (+) and Lopinavir/Ritonavir and the correct abbreviation LPV/r

***Response***

Line 189-"MTB/Rif" now appears in full the first time it appears in the text

Line 192-“PTB” now appears as “Pulmonary TB” the first time it appears in the text

Line 224-“PMDT" written in full the first time it appeared in line 149.

Line 230-233- Lopinavir/Ritonavir) (ABC + 3TC + EFV now reflected and LPV/r now reflected

**Comment #6**

6. The authors have introduced selection bias, which is a limitation of the study. The study includes MDR/RR-TB patients who initiated treatment between 2010 and 2015 and continued treatment at either district hospitals or urban polyclinics (Line 237). Those who were referred to primary health facilities were excluded (Line 239). This may contribute to why treatment outcomes are different to what is reported to the WHO by the NTP of Zimbabwe. Those who are referred to primary health facilities are considered more stable, tolerating the regimen, and live close to the PHC. These factors, including distance, have been shown to be associated with treatment outcomes, adherence to treatment and utilization of treatment services. The study population therefore presents patients who were unstable/not tolerating the regimen and/or those who lived >10km from the health facility. The fact that the study does not include any treatment outcomes for patients referred to primary health facilities is a limitation.

***Response***

Thank you very much for concurring with the authors that the study had some limitations as referred above. The above Editor concern has been articulated at length as a study limitation and authors acknowledged this quite extensively

**Comment #7**

7. Line 219 - please clarify what constitutes "stable". What criteria is used to determine if a patient is stable?

***Response***

“Stable” is a term used to describe those patients who were able to ingest medication, did not show signs of adverse drug reaction and had all the laboratory investigations within normal limit. This description now appears in line 219

**Comment #8**

8. Line 252 - Please clarify how "missed doses" is reported. Is this patient self-report or obtained from clinical data (e.g. dispensed)?

**Response**

“Missed doses” are determined from patient clinical records specifically from the patient treatment card. As the directly observed treatment is provided, the ‘right tick’ is made against the particular date in the treatment card by the clinician when drugs are consumed. In case if the patient fails to report for DOT at the health facility, a follow up is done and in the event the patient is not located and misses his drugs for that day, it is marked as missed with ‘wrong’ tick against that date. We considered all the ‘wrong’ tick till the date of treatment outcome as ‘Missed doses’, Later we calculated the percentage of missed doses per total number of days the patient was receiving the treatment.

**Comment #9**

9(1) Line 269 - regarding treatment outcomes; (1) authors should refer to the WHO reporting framework and include the definitions of the outcomes.

***Response***

Noted. “Table of Treatment Outcomes definition” referred as Fig 2

9(2) authors needs to include when the outcome was defined (i.e. how long were patients followed-up for, what is the person-time?), (3) the definition of primary outcome seems unusual - can authors include a reference for this?, (4) since death and LTFU are assigned when the outcome occurs, it is more appropriate to combine death + LTFU in your outcome definition and use Cox Proportional Hazard regression

***Response***

Patient TB treatment outcomes were defined at 24 months from date of each patients MDR-TB treatment start date until date of their respective outcomes for deaths, LTFUs, treatment failure and “outcome not evaluated”. Those classified as LTFU were defined as a patient whose MDR-TB treatment was interrupted for two or more consecutive months for any reason as per WHO guidelines. Those cured or who completed their treatment were categorized as successful treatment outcomes, whereas the others were categorized as unsuccessful treatment outcomes in line with recent meta-analysis studies by Kibret et al and Johston JC et al. We have added this to the data analysis section of our paper

The 473 patients in our study contributed 672.5 person-years. We have added this to the first paragraph of the results section. Thank you for suggesting that we use Cox Proportional Hazards regression. We considered this at length as the most appropriate analysis method, however we finally abandoned it because there were a number of patients who had missing dates on treatment outcomes hence this would lower our sample size.

**Comment #10**

10. Line 280 - 282 - IRB protocol numbers need to be included. Line 283 - "health and child care" should be corrected as in Line 282 "Ministry of Health and Child Care"

***Response***

Line 280 - 282 - IRB protocol numbers now included.

Line 283 - "health and child care"- Both sentences line 282 and 283 read as “Ministry of Health and Child Care” from original texts

**Comment #11**

Line 233 - Guidelines for the use of cotrimoxazole (CPT) should be included in the Methods.

***Response***

A reference for the guidelines of use of Cotrimoxazole Preventive Therapy is now indicated and the reference section has been updated

**Comment #12**

12. Line 569 - some key variables are missing in Table 2. Examples include resistance pattern (e.g. MDR-TB, RR-TB (mono; isoniazid sensitive) or RR-TB with additional resistance unknown), time on ART, CD4 count, smear microscopy results, weight/BMI (Line 268), EPTB vs. PTB.

***Response***

Thank you for observing that some key variables are missing in Table 2. All the above are missing data as explained in the section on study limitations

**Comment #13**

13. Line 236 and Table 1 - please clarify if the study includes children? From Table 1 n=24 were <24 years. It is not clear if this is 18-24 or 0-24? If the latter, then this should be further categorized as children (<10) or adolescents (10-24) or further as young adolescent (10-14), older adolescent (15-19) and young adult (20-24).

Response

The study does include children. We had collapsed the age groups <24 years because of small numbers, however we have reverted back to the disaggregation of <5, 5-14 and 15-24 years in Table 1 but however maintained the combined age group of <24 years in the multivariate regression analysis table so as to allow for adequate numbers for calculating relative risks and their 95% confidence intervals. We also used the age categories <24, 25-34, 35-44, 45-54 and 55+ years as these are the standard age groups for reporting TB data to WHO and are also used in several studies to allow for comparisons.

**Comment #14**

14. Table 2 Encountered SAEs - Is this during treatment or at treatment initiation. If the former, then this should be separated out from the characteristics at treatment initiation (Clarify that Table 2 includes clinical characteristics AT treatment initiation). Would be useful to include/mention the most common SAEs reported in the Table (i.e. Table 3?)

**Response**

Thank you for the point. The encountered SAEs were during treatment and we have rephrased the variable as “*Encountered SAE during treatment*”. Seeing that some patients may have also initiated ART during MDR-TB treatment and that we have the variable on missed treatment dosses in this table, we have instead rephrased the table title as “***Clinical characteristics of MDR/RR-TB patients at baseline and/or during treatment among MDR/RR-TB patients initiated on treatment in Zimbabwe, 2010-2015***”. We hope that this is satisfactory.

**Comment #15**

15. Table 3 Outcomes should add up to 100% - Failed should be 0.9% and not <1 and LTFU 8.2% (39/473). Therefore, 26.4 + 8.2 + 0.9 + 3.4 = 100%

***Response***

Many thanks for pointing this out. We have replaced the percentage for those with treatment failure with 0.8% however we note that the percentages for the different end-of-TB treatment outcomes add up to 99.9% due to rounding-off errors. We have specified this as a footnote to the table.

**Comment #16**

16. Table 4 - consider defining a resistance pattern variable - which should encompass the diagnostic test and the DST results.

***Response***

Thank you for the comment. This information was not available due to missing data on CDST results which the authors highlighted as a study limitation in the discussion section.

**Comment #17**

17. Table 4 – The current model ignores the time-varying nature of adherence.

***Response***

This is indeed a valid comment and we ought to have included missed doses as a time dependent variable in our univariate and multivariate-adjusted regression models. However, we did not have data on the specific time points when MDR-TB treatment doses were missed as these were aggregated for each patient by the time they achieved their treatment outcome

**Comment #18**

18. Figure 1 - It would be useful to present outcomes as typically reported by the WHO (e.g. see [https://www.who.int/tb/publications/global_report/gtbr2017_main_text.pdf - Page 80](https://www.who.int/tb/publications/global_report/gtbr2017_main_text.pdf%20-%20Page%2080))

**Response**

Thank you for the comment. The term “Unfavourable Outcome” has been further clarified in the text as per WHO reporting system (Death, Treatment failure, LTFU and Not Evaluated)

**Reviewer #1:**

I appreciate the work of the Authors to try to define factors associated with unfavourable outcomes in RR and MDR TB in a Country with a very high percentage of HIV co-infected patients.
I suggest the Authors to stress the need for individualized treatment regimens in order to decrease side effects (BMC Infect Dis. 2019 Jun 28;19(1):564. doi: 10.1186/s12879-019-4211-0.) and to cite the need for better treatment compliance (BMC Infect Dis. 2017 Jan 21;17(1):91. doi: 10.1186/s12879-017-2200-8. and Multidiscip Respir Med. 2018 Nov 9;13:41. doi: 10.1186/s40248-018-0154-3. eCollection 2018)

**Response**

The authors appreciate this valid comment. On study implications, line 409, the authors stresses the need for NTP to adopt shorter treatment regimen for MDR-TB treatment which has been shown to have fewer SAEs. A reference has been added and cited in the reference section as advised
Line 417, the authors stresses the need to ensure that patients do not have missed doses during MDR-TB treatment in order to lessen their risk of having unfavourable outcomes. . A reference has been added and cited in the reference section as advised.

**Reviewer #2:**

**TITLE** – Consider adding ‘Urban Setting’ to the title since the population belongs to urban areas and probably a bias to favorable outcome.

**Response**

Thank you for raising this important point, however the study was not primarily set for the urban population as some district hospitals serve mostly rural populations thus the study population was both urban and rural

**Abstract** – appropriate – consider elaborating the methods.

***Response***

Thank you for this comment. The methods section of the abstract has been elaborated as advised. The following statement has been added “A generalized linear model with a log-link and binomial distribution or a poisson distribution with robust error variances were used assess factors associated with “unfavorable outcome”. The unadjusted and adjusted relative risks were calculated as measure of association. A 𝑝value< 0.05 was considered statistically significant”.

**Introduction** – Describes the problem in a complete and relevant manner. Aim of the study is well reported.

***Response***

Thank you, well appreciated

**Comment**

**Method –** Please consider mentioning the type of phenotypic cultures utilized.

Response

Thank you. The type of phenotypic cultures utilized is now indicated as BBL^TM^ MGIT^TM^ Mycobacterial Growth Indicator Tubes (Becton Dickinson, Sparks, MD

**Comment**

Operational definition of treatment outcomes, although standardized as per WHO – please reiterate here, this can prevent a bit of confusion

***Response***

Noted, Fig 2 has been added to clarify

**Comment**

Results – Now as mentioned by the authors in terms of limitation – the absence of data on management of SAE, CDST results, CD4 count and comorbidity data. I can suggest certain key data variables which are missing, if available, consider providing them.
BMI, radiological evidence of cavities as they may have direct effect on outcome as well as occupational history and smoking status of the cohort, both of which are strong risk factors for developing TB and unfavorable outcome

***Response***

We thank you for raising this very valid comment. As already mentioned as study limitations, the data suggested was missing and cannot be included

**Comment**

It is unfortunate that DST results of fluoroquinolones and second line injectables are not available, both of which are known to be risk factors for adverse outcome, if for the ~53 cases this data can be made available and analysed as a risk factor, it will greatly strengthen your study. It is a strong point for advocating the need of DST.

***Response***

Thank you for the comment. Of all the second-line injectables and flouroquinolones in use (*i.e. kanamycin, Levofloxacin, Moxifloxacin and Cycloserine and Capriomycin alone as an alternative to those intolerant to kanamycin*), only 3 patients were resistant to any one of the above hence we could not include this variable in the univariate and multivariate regression analysis because of the small numbers.

**Comment**

Data for Time to unfavourable outcome – is a strong variable that can help us in understanding the severity of the cohort population and help in future intervention. Did any SAE led to unfavourable outcome? The individuals who died or lost to follow up – were recorded to be failing the regimen?

***Response***

Thank you for raising this very important observation. In our univariate and multivariate regression encountering an SAE was not significant predictor of an unfavourable outcome as shown in Table 5. Also, due to deficiencies in our data and this being a retrospective study, we failed to assess the cause-effect relationship of SAEs and unfavourable outcome

**Comment**

Details of ART initiation is not provided neither the status of ART success or failing is analysed.

Response

Thank you. Unfortunately data on virological, immunological or clinical failure of patients initiated on ART were not available in the DR-TB registers hence required matching patient names to the ART registers given that there was no unique identifier linking the ART and MDR-TB programmes. However we do acknowledge the importance of this data and is something that was worth exploring and should be explored in future studies.

***Comment***

Discussion:

The authors have done proper analysis of their results, aptly described the limitation and strengths of the study as well as compared the results with similar studies.

***Response***

Thank you, comment well appreciated

**Comment**

Please elaborate on the reason for 180 people with no recorded time for culture conversion – they failed to culture convert or some of them died before or some could not give the sample

***Response***

Thank you for asking us to elaborate. These patients either did not have their samples examined because of contamination, spillages or did not have results (CDST) sent back to the health facility and the reasons are well explained under the section on study implications where the authors mentioned that the high proportion of patients who did not have CDST results during their treatment is cause for concern as this is essential in monitoring bacteriological response to treatment. A recent study from Zimbabwe showed leakages in receipt of sputum samples at NRLs, culture contamination among received sputum specimens leading to a reduced proportion of samples with CDST results and this was mentioned in the relevant section. The authors concluded by stressing that this CDST system will require improvements including feedback of CDST results to facilities in order to inform patient management.

**Comment**

How many patient had their sputum culture status reverted back to positive

***Response***

Thank you for this question. None had their sputum culture status reverted back to positive

**Comment**

Interestingly, ~14% of the study cohort was initiated on ATT beyond 30 days of RRTB diagnosis, was not found to affect the outcome. You may consider highlighting that delay in initiation for SLD.

***Response***

Thank you for pointing this out. We have mentioned the following in the discussion section: *“Last, a significant number of patients delayed initiation of MDR-TB treatment by more than 30 days after RR-TB diagnosis. Whilst this was not associated with having an unfavourable outcome, this has got dire consequences at an individual level towards disease progression and at a population-level towards MDR-TB transmission in the community.”*

**Reviewer #3:**

**Comment #1**

Background is unclear and does not adequately contextualize the aims of the manuscript.

***Response***

Thank you for the comment. This study aimed at assessing the profile, treatment outcomes and factors associated with unfavourable treatment outcomes among patients initiated on MDR/RR-TB treatment. In view of this we started by highlighting the global burden of MDR/RR-TB and also mentioned the WHO MDR/RR-TB treatment targets as a benchmark to assess our poor performance in this regard. We further summarised some of the literature of risk factors associated with poor MDR-TB treatment outcomes before we highlighted the paucity of data on treatment outcome determinants in our setting. This is of interest to the Zimbabwe NTP in order to have tailored interventions aimed at improving our overall MDR-TB treatment success rate. We do appreciate your comment and would like your further guidance on specific sections that require rewriting in order to make the introduction more appealing.

**Comment #2**

Methods lack clarity on how outcomes were defined, time-points at which outcomes were assessed by, measurement of covariates like adverse events is not suitable, inferential analytic techniques are also questionable.

***Response***

Thank you for this comment. We have addressed your comment on outcome definitions and time-points for assessment of these outcomes are addressed in the data entry and analysis section in line with other reviewers comments. As for inferential analytical techniques, we would have preferred to use Cox Hazards regression analysis to generate hazard ratios, however due to a large proportion of missing data on time of outcomes, we resorted to generating relative risk instead. Also, as our outcome of interest in this manuscript is ‘unfavourable outcome’, which includes ‘death’, LTFU, failure and ‘not evaluated’ we considered binomial regression. If at all we were modelling for death or LTFU than the Cox Hazard regression would have been the only option.

**Comment #3**

Results are sparse and could be described in greater detail.

***Response***

Thank you. In our analysis, we generated tables that responded to each of our set specific objectives and were hesitant to delve into greater detail for fear of digressing from our set objectives. However, we would appreciate if you can specify particular detail which would be of interest to the readers of our paper and is also aligned with our study objectives.

**Comment #4**

It is not very clear how background, aims, methods, results tie into the discussion and conclusions.

***Response***

Thank you for the comment. We are however surprised that this is the case as your comment is contradictory to the other 2 reviewers. In writing the paper we carefully attempted to logically align our discussion to the results presented which in turn were informed by the background, aims and methods of the study. We hope this is understandable with you

**Comments:**

1. Keywords
   a. Repeated (full-text and abbreviations used for the same word)

***Response***

We have gone through the whole manuscript and ensured that full-text and abbreviations are reported together only once, following which only abbreviations are mentioned were necessary.

a) **Background**

***Comment***

Tuberculosis to be written out in full before abbreviated

***Response***

Thank you. Tuberculosis now written out in full the first time it is used before being abbreviated

**Comment**

Incorrect order of abbreviations for RR/MDR-TB. Authors write this out in full starting with MDR-TB.

***Response***

Thank you for this. We appreciate that MDR/RR-TB can be written as RR/MDR-TB however after careful consideration, the study investigator team opted to align with MDR/RR-TB which is used in the WHO global TB reports and guidelines. We hope this is understandable.

***Comment***

Please rephrase last sentence. This is unclear

***Response***

Thank you for the comment. The last sentence on the background now rephrased and reads “The profile, management, and factors associated with unfavourable treatment outcomes of MDR/RR TB have not been systematically evaluated in Zimbabwe”.

**Comment**

b) Design

1) Please provide more detail regarding the methods you employ to assess treatment outcomes and risk factors for such outcomes. Study sites, participants, data collected, analytic methods used, definition of outcomes assessed, time point outcomes were assessed by etc.

**Response**

Study sites were all district hospitals and urban polyclinics in Zimbabwe. The study participants were all MDR/RR-TB patients initiated on treatment between 2010-2015 under the Zimbabwe NTP and continued their treatment in the above mentioned health facilities. This information is stated in the section on “Study Population”. We have further on added the total number of district hospitals and urban polyclinics which totalled eighty-two under study design. As per your earlier request we have specified the time point outcomes assessed in the “Data entry and analysis”

2) How were participants selected for the final analysis?

***Response***

We thank you for asking us to explain. Of the total 935 MDR/RR-TB patients initiated on treated during study reference period , the 473 (51%) of patients who met the study inclusion criteria of those started on MDR/RR-TB treatment and continued their 20-24 month treatment at district and urban polyclinics were all included in the study

**Comment**

c) Results

i. How was missing MDR-TB treatment doses defined? Please add to methods.

***Response***

Thank you. *Percentage of missed doses is defined as* percentage of the number of days with missed doses divided by the total number of days a patient was on treatment up until date of outcome. This has been added to methods section

**Comments**

ii. Did outcomes differ between RR-TB and MDR-TB?

***Response***

Thank you for this valid comment. As shown in Table 5, based on the individual analysis of isoniazid, streptomycin and ethambutol resistant patterns at baseline, there were no differences in outcomes between RR-TB and MDR-TB patients. Furthermore, it is difficult to conclusively distinguish between RR-TB and MDR-TB patients as the majority of patients did not have DST results recorded or at least for all second-line TB drugs..

Comment

iii. How were covariates selected to be included in the main effects model? Please add to methods.

***Response***

Thank you for the comment. All covariates with a p<0.25 and those that are biologically plausible predictors of unfavourable outcomes were added to the multivariate regression model. This is mentioned in the “Data entry and analysis” section.

**Comment**

iv. Why was ‘not evaluated’ outcome considered unfavourable?

***Response***

Those “not evaluated” were included in those with unfavourable outcomes as stated in the “Data entry and analysis” section in line with recent meta-analysis studies by Kibret et al and Johston JC et al. The WHO in its annual TB report, considers ‘not evaluated’ as ‘unfavourable’ or ‘unsuccessful’ outcome

**Comment**

d)Conclusion

v. Stating that outcomes were poor coupled with high occurrence of adverse events make it seems like this was a significant risk factor – but it wasn’t?

***Response***

Thank you for the comment. Whilst we acknowledge that adverse events were not a significant risk factor, in paragraph 6 of the discussion section we only highlight that the high proportion of SAEs in this patient cohort (i.e. one in four patients) is a cause for concern and do not insinuate that this is a risk factor for unfavourable outcomes. We further advocate for more standardized recording and reporting of SAEs for better tracking by the NTP and also advocate for wider adoption of the shorter MDR-TB treatment regimen

***Comment***

vi. Conclusions and recommendations are based on adverse events; however this is not mentioned as a significant predictor in the results. Therefore, how are these conclusions relevant to your findings?

***Response***

*Thank you. Please refer to the earlier response above.*

2. Introduction -Comment
   i. Capitalize ‘tuberculosis’ line 112

***Response***

“T” Now in capital letters

***Comment***

ii. Don’t start a sentence with an abbreviation – line 114 and other places within the text

***Response***

Line 114 and 118 corrected

***Comment***

*Insert “The” before World Health Organisation*

***Response***

The World Health Organization’ – line 116

“The” Inserted

**Comment**

iii. Line 120 – is this reference from 2000?

***Response***

Thank you. Yes, we can confirm that the reference is from 2000

***Comment***

iv. Line 128-129 – please clarify what the second part of this sentence means

***Response***

The authors of the systematic review paper stated that studies included in the review were largely from countries with low HIV coinfection. They then recommended for systematic assessment of treatment outcomes in high HIV coinfection countries of sub-Saharan Africa

**Comment**

v. Could you comment on the incidence of ADRs on second-line TB treatment? Line 130-134

***Response***

Thank you for raising this comment. We could not generate incidence rate of ADRs as we did not have data on time of their occurrence.

**Comment**

vi. Could you comment on eligibility for newer shorter regimens? Line 130-134

***Response***

Thank you for asking us to comment. Eligibility for newer shorter regimens is when there is no risk or confirmed resistance to any second line medicines or any medicine used in the shorter treatment regimen

**Comment**

vii. However, unsure of commenting on newer short course regimens – what does this have to do with assessing treatment outcomes in your traditional-long course cohort? Shorter regimens may be more pertinent in your discussion.

***Response***

Our apologies as we do not clearly understand your comment. If we understand well, you suggest that we mention the importance of the newer short course regimen as an alternative to the traditional long course regimen. We have touched on this in paragraph 10 of the discussion section were we suggest that the NTP should adopt wider scale up of the shorter treatment regimen which has fewer SAEs although treatment outcomes are comparable to the long MDR-TB treatment regimen.

3. Methods

**Comment**

viii. Could a figure describing the general setting be included to better understand the public healthcare setting? And thereafter selection process

**Response**

Thank you for suggesting this. We have attempted to present this information graphically but have concluded that the text description mentioned in the General setting and the description of diagnosis and treatment of MDR/RR-TB gives an overall description of Zimbabwe’s public health setting in the context of the National TB Programme. We are happy to clarify any specific sections that are unclear to you.

**Comment**

ix. Line 212-213 – WHO recommended DOTS-plus during the study period? Or as above in 2000?

***Response***

In 2000, Tense changed to reflect “Was” instead of “be”

**Comment**

x. Line 237 -238 please rephrase

**Response**

Rephrased as per reviewer 3 comments, sentence now short and more precise

**Comment**

xi. Would patients referred for DOTS-plus who were then excluded from the analysis be systematically different to those who were included?

***Response***

Yes we do acknowledge this possibility given that the treatment success rate for our study population was better than that reported by the NTP for all patients due to their high proportion of unevaluated patients. Furthermore in rural settings health facilities are less accessible due to long distances from households and there is less specialized care in rural facilities. We have stated this as a limitation in the discussion section.

**Comment**

xii. Line 243 – consistency with capitalization

**Response**

We have noted this and addressed it accordingly.

**Comment**

xiii. Data quality between sites could differ? Were there any systematic differences between quality (missing data) etc. between sites?

**Response**

Thank you for this comment, however there were no systematic differences between sites in terms of data quality. Missing data was across all sites because the tools used by all sites were the same and no data was collected on those variables. Also all the sites sent their samples to the same reference laboratory which did not give results back on CDST. Furthermore 10%of the data was subjected to quality assurance and originally missing data was completed before final data entry.

**Comment**

xiv. Since the primary outcome of interest is death and LTFU which can occur any time from treatment initiation – would you consider using a Cox Hazard Model to determine hazard ratio estimates of ‘unfavourable outcomes’? Considering time to outcome may be an important factor which could be affected differently by different risk factors. Furthermore, was LTFU and death separated and did risk factors differ by these outcomes? Lastly, what is the motivation for including ‘not evaluated’ as an ‘unfavourable outcome’?

**Response**

As mentioned above we could not perform Cox Hazard regression analysis due to the high proportion of missingness on time to event data. We also reserved further comparisons on any differences between predictors of mortality and LTFU for another paper as we felt this was too much information for one paper. We hope reviewers understand our position.

**Comment**

xv. Please include a time-point at which outcomes were assessed by. Did everyone have a minimum follow-up etc.?

**Response**

Yes, since this was a retrospective cohort study based on routinely collected programme data, all patients were allowed a follow-up period for their potential 20-24 month MDR-TB treatment duration.

**Comment**

3Results

xvi. Line 287 – punctuation

**Response**

Thank you, we consider the punctuation used to be fine and appropriate as “Ethics approval was granted by The Union Ethics Advisory Group of the International Union against Tuberculosis and Lung Diseases, Paris, France, IRB number EAG 53/18 and the Medical Research Council of Zimbabwe (MRCZ), IRB number MRCZ/A/2331”

**Comment**

xvii. Measurement of adverse events seems to have been recorded throughout treatment for respective patients. If so, this cannot be included as a potential risk factor in the predictive analysis as results would be prone to systematic bias. Those who remain in care long enough to develop and record ADRs will be different from those who do not. If ADRs are to be included, this should be recorded within a pre-defined period around baseline i.e., within 14 days after starting treatment for example. Thereafter, the analysis should be rerun.

**Response**

Thank you for the valid comment. We have removed SAEs from the multivariate regression model.

**Comment**

xviii. Table 4 – stepwise approach to model selection given in methods – but no p-values shown in table?

**Response**

Thank you for raising this comment. We did not include p-values as they would be redundant given that the reported univariate and multivariate-adjusted relative risks with 95% confidence intervals are more informative. When the 95%CI doesn’t cross the null value, then the factor is significant.

**Comment**

xix. How would the reader interpret significant risk factors for variables with categories that were ‘not recorded’? i.e., streptomycin DST pattern, Ethambutol DST pattern? Isoniazid DST pattern, missed doses?

***Response***

We appreciate your comment, however the interpretation for unrecorded data will be largely not make programmatic interpretation especially for the “missed doses” variable. We however included all the patients with unrecorded data in the univariate and multivariate-adjusted regression models for the selected variables since excluding them will greatly diminish the eligible sample given the large amount of missing data as is often the case with routinely collected programme data.

***Comment***

3. Discussion
   i. Line 316-324 – please rephrase language used here.

***Response***

Thank you. Rephrased to make the statement clearer as” The proportion with an unfavourable treatment outcome (death, Treatment failure, LTFU and Not evaluated) increased annually from 0% in 2010 to 45% in 2015 (**Figure-1**).

Factors associated with an unfavourable outcome among patients on MDR-TB treatment are shown in **Table 4**.

Those who were HIV-positive and ART-naive (ARR=2.83; 95% CI: 1.44-5.57) and those who missed >10% of their MDR-TB treatment dosses (ARR=2.41; 95% CI: 1.59-3.67) were more likely to have an unfavourable treatment outcome

**Comment**

ii. Line 325-326: what was the breakdown of patients across sites? Did some sites have a disproportionally higher number of patients?

***Response***

*Thank you for the comment. Fifty-two out of sixty-three district hospitals that notified an MDR/RR TB patient during 2010-2015 contributed different numbers of the patients included in the study who were not refered to rural health centres. Thirty polyclinics from the two metro-politan provinces also contributed varied numbers but less patients per site as compared to what the district hospitals notified.*

*Across sites, yes the numbers were different depending on various factors as per the inclusion criteria described in the study design.*

**Comment**

iii. Please consider the use of language in the strength and limitations sections of the discussion.

**Response**

The language used is deemed appropriate for a scientific paper as it arranges the strengths and limitations as first, second, third etc thus allowing the reader to follow the sequence of the issues highlighted.

**Comment**

iv. Line 328 – how were operational challenges assessed in this analysis?

**Response**

Since this study was based on a retrospective review of routine patient records. Therefore we did not conduct a process evaluation which would have been useful in determining operational challenges encountered in treatment and management of patients on MDR-TB treatment

**Comment**

v. Line 336 and 337 seems to be in contradiction to line 325-326

***Response***

Thank you for the observation, we however think there is no contradiction as a district has both urban and rural population. The participants included in our study were predominantly from the urban part of the district yet those that were excluded were predominantly from the rural part of the same district. It is not necessarily correct to assume that districts have only rural population

**Comment**

vi. Line 343-344 – if these changes were assessed during treatment, I’m not sure they would be suitable in your predictive analysis for the reasons of systematic bias as mentioned above

***Response***

*Thank you****.*** Comorbidities were determined during eliciting patient history at MDR-TB treatment initiation. We have revisited line 343-344 and specified this.

**Comment**

vii. Line 357-359 – could you comment on time on ART

***Response***

Thank you. unfortunately data on ART initiation start dates were not available in the DR-TB registers hence we could not assess timing of ART in relation to MDR-TB treatment commencement. We have added this as a limitation to paragraph of the discussion section.

**Comment**

viii. Line 363 – still unsure of exactly how missed doses was defined? Please could this be clarified in the methods.

***Response***

Thank you, Missed doses are clearly defined under Methods (Operational Definitions) Line 255 as percentage of the number of days with missed doses divided by the total number of days a patient was on treatment up until date of outcome.

**Comment**

ix. Line 387 – at what time-point was cured/completed outcomes assessed? Was this by 24 months on treatment? As mentioned above – the time point at which outcomes were assessed is not clear here.

**Response**

Thank you. Time point of cured/completed outcomes was assessed at the end of treatment duration.

**Comment**

x. Line 411 – could you provide a measure of time between missed doses in the results section.

***Response***

Thank you. The following statement has been added to the results section under implications of study results: “ If a patient consecutively misses doses of two months or more, the patient is considered lost to follow-up and will restart treatment upon return”.

***Comment***

xi. Line 419-423 – it is still not clear how findings presented here link to the conclusions presented.

***Response***

Our conclusions were linked to our major study findings which are as follows: 1) the MDR-TB treatment success rate was lower than the WHO treatment success target 2) treatment outcomes were better among those on ART whilst 3) missed doses of more than 10% were associated with poor outcomes. In addition, we have also made mention of the high occurrence of SAEs

We thank Reviewer 1, 2 and 3 including the Editor for a thorough review and hope we have satisfactorily addressed all the grey areas. We are more than pleased to attend to any further comment that may require a follow-up response
