# Supplementary material for: Treatment outcomes of multi drug resistant and rifampicin resistant Tuberculosis in Zimbabwe: A cohort analysis of patients initiated on treatment during 2010 to 2015
Source: PLoS One. 2020 Apr 30;15(4):e0230848. doi: 10.1371/journal.pone.0230848 (PMC7192497; doi:10.1371/journal.pone.0230848)
Supplement: Attachment — Submitted filename: Plos One - Second Round Comments.docx [file pone.0230848.s003.docx]

# General Comments:

1. A language editor is strongly recommended. Acceptance of this manuscript should at the very least be contingent on this.
2. Have the authors looked into the rates and risk factors of unfavorable outcomes separately? i.e. LTFU vs. all-cause mortality vs. unreported etc.? Risk factors may differ across these outcomes and a stratified analysis may add more granularity to this paper (authors should consider at least having these separated outcomes in supplementary tables).
3. Would the ‘not evaluated’ outcome include those patients who were still alive and in care by 24 months who had not completed treatment? These patients could be different to those who have met the definition for LTFU, which speaks to the point above.
4. Gender breakdown listed in abstract is different from Results section. Keeping the reference category the same may make the manuscript easier to read.
5. Missing >10% of treatment doses seems to be ascertained throughout treatment and not a once off measurement at baseline? If this is the case, then this **cannot be used as a predictor of unfavorable outcome**. “*Percentage of missed doses:* percentage of the number of days with missed doses divided by the total number of days a patient was on treatment up until date of outcome.”
6. “study findings were reported in accordance with Strengthening the Reporting of Observational Studies in Epidemiology (STROBE) guidelines.” This should be included in the methods and not discussion.”
