# Supplementary material for: Treatment outcomes of multi drug resistant and rifampicin resistant Tuberculosis in Zimbabwe: A cohort analysis of patients initiated on treatment during 2010 to 2015
Source: PLoS One. 2020 Apr 30;15(4):e0230848. doi: 10.1371/journal.pone.0230848 (PMC7192497; doi:10.1371/journal.pone.0230848)
Supplement: Attachment — Submitted filename: R2 Response to reviewers.docx [file pone.0230848.s004.docx]

**Response to Reviewers**

**Rebuttal Letter**

PLOS ONE

The Editor in Chief

23 December 2019

*REF: PONE-D-19-16150: Treatment outcomes of Multi drug resistant and Rifampicin resistant Tuberculosis in Zimbabwe: A cohort analysis of patients initiated on treatment during 2010 to 2015*

Dear Editor

Thank you very much for reviewer comments and suggestions during second round review of our above named manuscript. We have carefully read through the reviews and revised our paper accordingly. We feel that the paper is much improved as a result of this review process and thank you for taking it to this stage. We have responded point by point to the comments and suggestions of the reviewers.

We would like to submit the revised version of the manuscript with highlights (R2- track) and the revised and clean version of the manuscript (R2-Clean). The line numbers in the response to reviewer comments corresponds to the line numbers in the revised version with track changes.

Sincerely

R. Matambo (Corresponding Author)

**Reviewer-1**

**Comment 1:** Consider adding – lack of radiological data in the heading of limitation.

**Author Response**
Thank you for your suggestion. We have added the statement under study limitations and now the paragraph reads as follows: “Second, there were missing data on key variables which include CDST results, socioeconomic status, WHO clinical staging, CD4 cell count, timing of ART in relation to MDR-TB treatment commencement, nutritional status, MDR-TB drug regimens and their dosages, data on virological, immunological or clinical failure of patients initiated on ART and radiological findings of TB lesions – all which are important factors which may be related to MDR-TB treatment outcomes”. Line 380

**Comment 2:** Clarification needed for patients – Lost to follow up or died- were the failing on the current regimen.

**Author Response**

Thank you for raising this important query.

As this was a retrospective study, we don’t have data to safely conclude whether these patients were failing on the current regimen. Of the 164 patients who died or were LTFU, the majority 112 (68.3%) had no culture conversion result recorded. Of the remaining 52 (31.7%), 48 (94.2%) had culture conversion within 6 months of initiating MDR-TB treatment. Thus, we have not tried to interpret these findings. However, we acknowledge this as a deficiency of the current study.

**Comment 3:** Details of ART initiation is not provided neither the status of ART success or failing is analysed. Please consider mentioning this shortcoming in the Discussion.

**Author Response**

Thank you for suggesting that we mention in the discussion section about the un-availability of data on virological, immunological or clinical failure of patients initiated on ART. This has now been mentioned in line 370-371

**Comment 4:** “study findings were reported in accordance with strengthening the Reporting of Observational Studies in Epidemiology (STROBE) guidelines.” This should be included in the methods and not discussion.”

**Author Response**

Thank you for such valid suggestion. We have since incorporated the changes as advised. Line 287-288

**Comment 5:** A language editor is strongly recommended. Acceptance of this manuscript should at the very least be contingent on this.

**Author Response:**

Thank you for this suggestion. We have engaged a well experienced scientific editor who has gone through the manuscript and made grammatical changes to the manuscript. The grammatical changes have been made throughout the manuscript.

**Reviewer 2:**

**Comment:** Have the authors looked into the rates and risk factors of unfavorable outcomes separately? i.e. LTFU vs. all-cause mortality vs. unreported etc.? Risk factors may differ across these outcomes and a stratified analysis may add more granularity to this paper (authors should consider at least having these separated outcomes in supplementary tables).

**Author Response:**

Thank you for the comment. You raise a very valid point. However, we have another paper in which we had more granular analysis on rates and risk factors of specific unfavorable outcomes (LTFU and death) which if included in this paper would result in duplication. We hope that this is acceptable with the reviewer and the editor if this further analysis is not included in this manuscript.

**Comment 2:** Would the ‘not evaluated’ outcome include those patients who were still alive and in care by 24 months who had not completed treatment? These patients could /be different to those who have met the definition for LTFU, which speaks to the point above.

**Response:** Thank you for the comment. None of the patients in this cohort were still on the treatment at the time of censoring date. Thus, we have not considered anyone still on treatment as ‘not evaluated’. Within the TB programme in Zimbabwe, MDR/RR-TB patients who would have completed their MDR-TB treatment with 1) a negative bacteriological confirmation result are considered cured whilst 2) those without bacteriological confirmation are classified as treatment completed. In response to your question above, only 3/33 (9.1%) were LTFU after being on treatment for 24 months. This implies that they were LTFU before completing their extended treatment duration which was a result of having missed treatment for a duration(s) not exceeding 2 months.

**Comment 3:** Gender breakdown listed in abstract is different from Results section. Keeping the reference category the same may make the manuscript easier to read.

**Author Response:**

We agree with your comment. We have therefore changed the narrative in the results section to match the reference category in the abstract i.e. “*….230 (49%) were males*”. Line 324-325

**Comment 4:** Missing >10% of treatment doses seems to be ascertained throughout treatment and not a once off measurement at baseline? If this is the case, then this **cannot be used as a predictor of unfavorable outcome**. “*Percentage of missed doses:* percentage of the number of days with missed doses divided by the total number of days a patient was on treatment up until date of outcome.”

**Author Response**:

Thank you for highlighting the deficiency of this variable. We acknowledge that this cant be used as a predictor. Hence, we have removed variable on missed doses from our univariate and multivariate regression model. We have also removed narrative in the abstract, results section and discussion section with reference to this predictor. Lines 111-112; 349-350; 362; 406-409; Table 5.

**Comment 5:** “study findings were reported in accordance with strengthening the Reporting of Observational Studies in Epidemiology (STROBE) guidelines.” This should be included in the methods and not discussion.”

**Author Response**

Thank you for such valid suggestion. We have since incorporated the changes as advised. Changes made in line number 370-371

We thank you for your esteemed review of our manuscript and hope that we have responded satisfactorily. Please do not hesitate to contact us for further clarification as your reviews enriches our manuscript.
