# Supplementary material for: Treatment outcomes of multi drug resistant and rifampicin resistant Tuberculosis in Zimbabwe: A cohort analysis of patients initiated on treatment during 2010 to 2015
Source: PLoS One. 2020 Apr 30;15(4):e0230848. doi: 10.1371/journal.pone.0230848 (PMC7192497; doi:10.1371/journal.pone.0230848)
Supplement: Attachment — Submitted filename: Reviewer Comments - Aug 2019.docx [file pone.0230848.s001.docx]

Mr Matambo and colleagues set out to assess treatment outcomes of RR/MDR-TB patients in Zimbabwe. A strength of this analysis is the use of a recent national cohort, outcomes of which have not previously been assessed. While this work is interesting and helps contextualize the burden in Zimbabwe, there are major concerns which need to be addressed before publication can be considered.

Overall comments that require attention:

1. Background is unclear and does not adequately contextualize the aims of the manuscript.
2. Methods lack clarity on how outcomes were defined, time-points at which outcomes were assessed by, measurement of covariates like adverse events is not suitable, inferential analytic techniques are also questionable.
3. Results are sparse and could be described in greater detail.
4. It is not very clear how background, aims, methods, results tie into the discussion and conclusions.

Comments:

1. Keywords
   a. Repeated (full-text and abbreviations used for the same word)
2. Abstract
   a. Background
      i. Tuberculosis to be written out in full before abbreviated
      ii. Incorrect order of abbreviations for RR/MDR-TB. Authors write this out in full starting with MDR-TB.
      iii. Please rephrase last sentence. This is unclear
   b. Design
      i. Please provide more detail regarding the methods you employ to assess treatment outcomes and risk factors for such outcomes. Study sites, participants, data collected, analytic methods used, definition of outcomes assessed, time point outcomes were assessed by etc.
      ii. How were participants selected for the final analysis?
   c. Results
      i. How was missing MDR-TB treatment doses defined? Please add to methods.
      ii. Did outcomes differ between RR-TB and MDR-TB?
      iii. How were covariates selected to be included in the main effects model? Please add to methods.
      iv. Why was ‘not evaluated’ outcome considered unfavourable?
   d. Conclusion
      i. Stating that outcomes were poor coupled with high occurrence of adverse events make it seems like this was a significant risk factor – but it wasn’t?
      ii. Conclusions and recommendations are based on adverse events; however this is not mentioned as a significant predictor in the results. Therefore, how are these conclusions relevant to your findings?
3. Introduction
   i. Capitalize ‘tuberculosis’ line 112
   ii. Don’t start a sentence with an abbreviation – line 114 and other places within the text
   iii. ‘The World Health Organization’ – line 116
   iv. Line 120 – is this reference from 2000?
   v. Line 128-129 – please clarify what the second part of this sentence means
   vi. Could you comment on the incidence of ADRs on second-line TB treatment? Line 130-134
   vii. Could you comment on eligibility for newer shorter regimens? Line 130-134
   viii. However, unsure of commenting on newer short course regimens – what does this have to do with assessing treatment outcomes in your traditional-long course cohort? Shorter regimens may be more pertinent in your discussion.
4. Methods
   i. Could a figure describing the general setting be included to better understand the public healthcare setting? And thereafter selection process
   ii. Line 212-213 – WHO recommended DOTS-plus during the study period? Or as above in 2000?
   iii. Line 237 -238 please rephrase
   iv. Would patients referred for DOTS-plus who were then excluded from the analysis be systematically different to those who were included?
   v. Line 243 – consistency with capitalization
   vi. Data quality between sites could differ? Were there any systematic differences between quality (missing data) etc. between sites?
   vii. Since the primary outcome of interest is death and LTFU which can occur any time from treatment initiation – would you consider using a Cox Hazard Model to determine hazard ratio estimates of ‘unfavourable outcomes’? Considering time to outcome may be an important factor which could be affected differently by different risk factors. Furthermore, was LTFU and death separated and did risk factors differ by these outcomes? Lastly, what is the motivation for including ‘not evaluated’ as an ‘unfavourable outcome’?
   viii. Please include a time-point at which outcomes were assessed by. Did everyone have a minimum follow-up etc.?
5. Results
   i. Line 287 – punctuation
   ii. Measurement of adverse events seems to have been recorded throughout treatment for respective patients. If so, this cannot be included as a potential risk factor in the predictive analysis as results would be prone to systematic bias. Those who remain in care long enough to develop and record ADRs will be different from those who do not. If ADRs are to be included, this should be recorded within a pre-defined period around baseline i.e., within 14 days after starting treatment for example. Thereafter, the analysis should be rerun.
   iii. Table 4 – stepwise approach to model selection given in methods – but no p-values shown in table?
   iv. How would the reader interpret significant risk factors for variables with categories that were ‘not recorded’? i.e., streptomycin DST pattern, Ethambutol DST pattern? Isoniazid DST pattern, missed doses?
6. Discussion
   i. Line 316-324 – please rephrase language used here.
   ii. Line 325-326: what was the breakdown of patients across sites? Did some sites have a disproportionally higher number of patients?
   iii. Please consider the use of language in the strength and limitations sections of the discussion.
   iv. Line 328 – how were operational challenges assessed in this analysis?
   v. Line 336 and 337 seems to be in contradiction to line 325-326
   vi. Line 343-344 – if these changes were assessed during treatment, I’m not sure they would be suitable in your predictive analysis for the reasons of systematic bias as mentioned above
   vii. Line 357-359 – could you comment on time on ART
   viii. Line 363 – still unsure of exactly how missed doses was defined? Please could this be clarified in the methods.
   ix. Line 387 – at what time-point was cured/completed outcomes assessed? Was this by 24 months on treatment? As mentioned above – the time point at which outcomes were assessed is not clear here.
   x. Line 411 – could you provide a measure of time between missed doses in the results section.
   xi. Line 419-423 – it is still not clear how findings presented here link to the conclusions presented.
